# Severe secondary hyperparathyroidism in a chronic kidney disease patient treated with Radiofrequency ablation: One case report

**DOI:** 10.3389/fmed.2022.876692

**Published:** 2022-07-22

**Authors:** Gulimire Muhetaer, Guangyi Liu, Ling Zhang, Hong Jiang

**Affiliations:** ^1^Xinjiang Clinical Research Center for Kidney Disease, Division of Nephrology, Department of Internal Medicine, People’s Hospital of Xinjiang Uygur Autonomous Region, Urumqi, China; ^2^Division of Nephrology, Department of Internal Medicine, Qilu Hospital of Shandong University, Qingdao, China; ^3^Division of Nephrology, Department of Internal Medicine, China-Japan Friendship Hospital, Beijing, China

**Keywords:** hyperplastic parathyroid gland, radiofrequency ablation, secondary hyperparathyroidism, chronic kidney disease, parathyroid hormone

## Abstract

End-stage renal disease (ESRD) is a global health problem with a high incidence (1) and a steadily increasing prevalence (2). Secondary hyperparathyroidism (SHPT) is a common and serious complication of chronic renal failure (CRF) in dialysis patients (3). It is mainly manifested as parathyroid hyperplasia caused by abnormal calcium and phosphorus metabolism and active vitamin D resistance, resulting in excessive secretion of parathyroid hormone (PTH), which leads to complications such as bone deformity, osteoarthralgia, pruritus, ectopic calcification, and cardiovascular calcification in CKD patients, significantly reducing the quality of life in CKD patients (4, 5). In patients with chronic kidney disease, secondary parathyroid gland hyperplasia needs to be treated as early as possible (6). Currently, there are a variety of treatment options, including vitamin D receptor agonists, xenacax hydrochloride, parathyroidectomy and ablation techniques, etc. (7, 8). Medical treatment is the main choice among these treatments, but it is invalid in patients with severe hyperparathyroidism. So, parathyroidectomy is suggested to do in those patients (9). However, many dialysis patients who have severe cardiopulmonary dysfunction cannot tolerate the trauma caused by surgery as the concept of minimally invasive surgery has been gradually introduced into all fields of surgery and medical treatment. Traditional surgery is no longer the only option. Radiofrequency ablation has been widely applied due to its advantages of less trauma, simple operation, and good repeatability. It has been reported to achieve good effects in treating secondary hyperparathyroidism patients (8). This case reports that one severe secondary hyperparathyroidism patient gets good therapeutic results from parathyroid radiofrequency ablation.

## Clinical data

**FIGURE 1:**
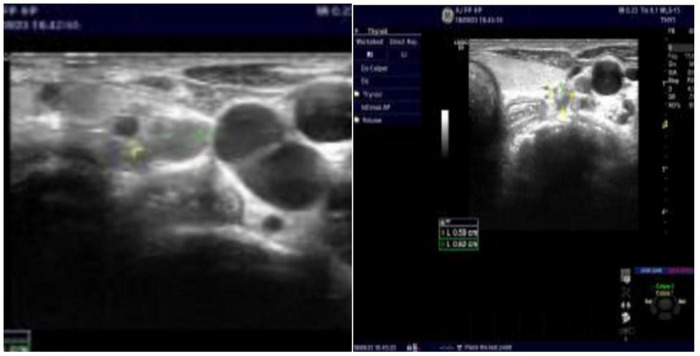
B-ultrasound image before parathyroid radiofrequency ablation.

**FIGURE 2:**
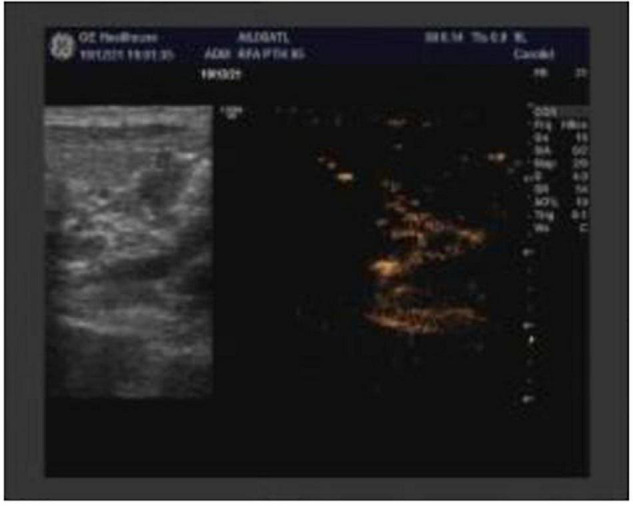
B-ultrasound image after parathyroid radiofrequency ablation.

This paper reported a case of secondary hyperparat hyroidism who underwent radiofrequency ablation to eliminate seven parathyroid glands hyperplasia. We report a 28-year-old male ESRD patient caused by primary glomerulonephritis, maintenance hemodialysis for 10 years, 3 times per week, intermittent bone pain for 2 years. Two years ago, he developed severe bone pain accompanied by a left-leaning spine and limited movement when sitting up and could not take care of herself. The blood level of PTH > 2500 pg/mL. Intermittent oral Rocaltrol implosive therapy and intermittent Cinacalcet therapy were used. His height was shortened by 28 cm in the past two years. A tumor appeared in the anterior part of the maxillary hard palate 6 months ago. The serum level of PTH > 2500 pg/ml, alkaline phosphatase 1284.00 U/L, calcium 1.99 mmol/L, inorganic phosphorus 1.54 mmol/L were rechecked. Five parathyroid tissues were detected by parathyroid B-ultrasound (Figure 1). The maximum one was 13 × 7.9 × 8.4 mm. He has a 10-year hypertension history, poor blood pressure control, admission blood pressure of 220/120 mmHg, a history of congenital heart disease, and a patent foramen ovale. The LVEF is 58%. Due to sequelae in 2006, children’s polio history underwent ’ left lower limb correction. CT findings: bilateral lung inflammation, heart shadow, pulmonary artery widening, pericardial effusion, thoracic scoliosis deformity. The pulmonary function examination showed severe restrictive pulmonary ventilation dysfunction. We conducted a multidisciplinary discussion. Refer to the guide, after discussion and communication, we decided to performed ultrasound-guided parathyroid radiofrequency ablation (bilateral) to this patient. In December 11, 2019, bilateral parathyroid radiofrequency ablation was performed. During the operation, local infiltration anesthesia was performed after dilution with 1% lidocaine, and physiological saline was used as isolation belt under the guidance of ultrasound to protect the trachea, skin and surrounding tissues. After that, the mass was isolated from the recurrent laryngeal nerve and bilateral paraproliferative glands were ablated by moving-shot ablation technique under the guidance of ultrasound, In addition to 5 parathyroid glands found by ultrasound, 2 parathyroid glands were detected buring surgery and total 7 parathyroid glands were eliminated (Figure 2), compression hemostasis was performed in the operation area. The patients without hoarseness, numbness of hands and feet, drinking cough and other discomfort complaints after operation. And the serum level of PTH reduced to 309 pg/mL. The paraproliferative gland was completely ablated reexamined by ultrasonography. After 3 days of symptomatic treatment such as calcium supplementation, the PTH level reduced to 96. 9 pg/mL, and discharged. During the follow-up, intermittent calcitriol, Caltrate and other medicines have been discontinued. On November 15,2021, the serum PTH level was 146.8 pg/ml, and the ALP level was 80 U/L. Bone pain disappeared. And the patients can basically stand on their own. The patient was followed up continuously.

## Discussion

SHPT is mainly characterized by calcium and phosphorus metabolism disorder, increased serum PTH secretion, and hyperparathyroidism. It is crucial to control the calcium, phosphorus, and PTH of patients with SHPT. The KDIGO guidelines suggest that PTH should be maintained at 2 ∼ 9 times the standard upper limit (9). It is recommended that patients with stage 5 CKD should use calcium analogs, calcitriol or vitamin D analogs, or combination treatment. This patient received intermittent administration of calcitriol and Sinakase oral. But the PTH level continued to rise. And multiple parathyroid glands were detected; the long diameter was more than 1cm of the largest one. Above mentioned reasons, combined with KDIGO’s recommendation, parathyroidectomy is recommended for CKD G3a ∼ G5D patients with severe hyperparathyroidism if medical treatment fails. However, many dialysis patients with cardiopulmonary dysfunction increase the risk of anesthesia complications and can’t stand the trauma caused by surgery. The clinical manifestations of this patient were bone pain, maxillary hard palate mass, shortening of height, and Poor cardiopulmonary function with malnutrition. Parathyroidectomy cannot be tolerated after evaluation, but traditional surgery is no longer the only option. Radiofrequency ablation has been widely used due to its advantages of less trauma, simple operation, and good repeatability. Radiofrequency ablation, laser ablation, and microwave ablation have been reported to have achieved good results in the treatment of secondary patients (8) and become an effective treatment method. Xu et al.’s study showed the iPTH and calcium levels controlled in 2 patients with SHPT by radiofrequency ablation (10). Although there are still controversies over surgical methods, efficacy, and safety, it is pointed out that there are still controversies over surgical techniques, effectiveness, and safety. However, radiofrequency ablation can also be regarded as an alternative therapy for patients with severe heart and lung function complications that increase general anesthesia risk and have achieved good efficacy (11). Therefore, we performed radiofrequency ablation for this patient. Seven parathyroid glands were eliminated during the operation. The PTH was also well reduced after the procedure. In SHPT patients, more glands may be affected, and the degree and number of glandular hyperplasia can seriously affect the prognosis of patients. The ectopic glands may exist in different locations, for example, the thyroid, cervical sheath, thymus, and upper mediastinum. Those glands cannot be approached by radiofrequency ablation adjacent to critical anatomical structures such as blood vessels or the esophagus. It will result in the failure of radiofrequency ablation and persistent SHPT after ablation. Studies have shown that the frequency of ectopic parathyroidosis in SHPT patients is about 15% (12). The proportion of patients with more than 4 glands is 2.5–30% (13). In this case, 5 parathyroids were detected by ultrasound. However, 7 parathyroids were seen in radiofrequency ablation operations. After another exploration, the ablated parathyroids were completely ablated. This shown that the positioning of instruments and doctors is particularly important, and experienced doctors can obtain better results through their own experience and repeated exploration to help find excess ectopic parathyroids. Alkaline phosphatase (ALP) is an indicator of osteogenesis, which reflects the high transport state of bone metabolism. Therefore, the higher level of ALP and the higher metabolic status of bone indicated the higher incidence of low level of serum calcium. Some studies have also pointed out that low calcium level after radiofrequency ablation is closely correlated with preoperative ALP (14). Higher ALP level before ablation will lead to more obvious postoperative hypocalcemia. In this patient, the level of ALP was very high before operation, and serum calcium decreased to 1.47 mmol/L after operation. Therefore, we actively supplement calcium and recheck PTH which also decreased compared with the first day after operation.

**FIGURE 3:**
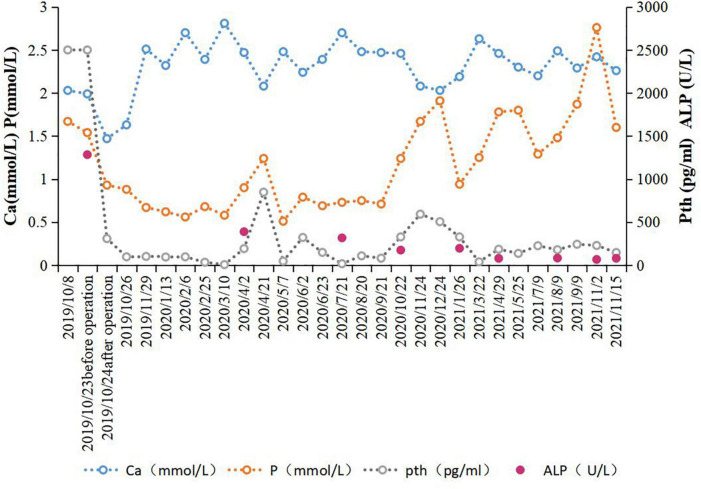
Relevant clinical test values before and after parathyroid radiofrequency ablation Reference values: Ca (2.11–2.52 mmol/L); P (0.85–1.51 mmol/L); parathyroid hormone (PTH) (12–65 pg/ml); alkaline phosphatase (ALP) (45–125 U/L).

Although there are still some differences in surgical methods, efficacy, and safety in the clinical guidelines and related studies, radiofrequency ablation is an effective treatment for patients with severe hyperthyroidism who cannot tolerate surgery. In this case, the patient’s bone pain disappeared after radiofrequency ablation. At present, the level of PTH is maintained between 136–242 pg/ml (Figure 3). The patient can stand on their own, and the quality of his life has been improved significantly, also very satisfied with this treatment. Based on this patient’s experience, we consider that when patients suffer from cardiopulmonary dysfunction due to basic diseases, and can’t perform or endure the trauma caused by surgery, radiofrequency ablation can be widely used because of its advantages of small trauma, simple operation and repeatability, the levels of serum iPTH, calcium and phosphorus can also be effectively reduced after surgery, so as to achieve the effect similar to surgical resection. Of course, it also requires longer follow-up and clinical indicators to comprehensively determine the clinical efficacy. Therefore, it is very important to choose the most suitable treatment according to the individual differences of patients when choosing treatment plans.

## Data availability statement

The original contributions presented in this study are included in the article/supplementary material, further inquiries can be directed to the corresponding author.

## Ethics statement

Written informed consent was obtained from the individual(s) for the publication of any potentially identifiable images or data included in this article.

## Author contributions

All authors listed have made a substantial, direct, and intellectual contribution to the work, and approved it for publication.

## Conflict of interest

The authors declare that the research was conducted in the absence of any commercial or financial relationships that could be construed as a potential conflict of interest.

## Publisher’s note

All claims expressed in this article are solely those of the authors and do not necessarily represent those of their affiliated organizations, or those of the publisher, the editors and the reviewers. Any product that may be evaluated in this article, or claim that may be made by its manufacturer, is not guaranteed or endorsed by the publisher.
